# Oxonium Ion Guided Analysis of Quantitative Proteomics Data Reveals Site-Specific *O*-Glycosylation of Anterior Gradient Protein 2 (AGR2)

**DOI:** 10.3390/ijms22105369

**Published:** 2021-05-20

**Authors:** Martina Pirro, Yassene Mohammed, Arnoud H. de Ru, George M. C. Janssen, Rayman T. N. Tjokrodirijo, Katarina Madunić, Manfred Wuhrer, Peter A. van Veelen, Paul J. Hensbergen

**Affiliations:** 1 Center for Proteomics and Metabolomics, Leiden University Medical Center, 2333 ZA Leiden, The Netherlands; M.Pirro@lumc.nl (M.P.); Y.Mohammed@lumc.nl (Y.M.); A.H.de_Ru@lumc.nl (A.H.d.R.); G.M.C.Janssen@lumc.nl (G.M.C.J.); R.T.N.Tjokrodirijo@lumc.nl (R.T.N.T.); K.Madunic@lumc.nl (K.M.); M.Wuhrer@lumc.nl (M.W.); P.A.van_Veelen@lumc.nl (P.A.v.V.); 2 Genome BC Proteomics Centre, University of Victoria, Victoria, BC V8Z 7X8, Canada

**Keywords:** glycoproteomics, TMT labeling, oxonium ion, LC−MS/MS, *O*-glycosylation, colorectal cancer, AGR2

## Abstract

Developments in mass spectrometry (MS)-based analyses of glycoproteins have been important to study changes in glycosylation related to disease. Recently, the characteristic pattern of oxonium ions in glycopeptide fragmentation spectra had been used to assign different sets of glycopeptides. In particular, this was helpful to discriminate between *O*-GalNAc and *O*-GlcNAc. Here, we thought to investigate how such information can be used to examine quantitative proteomics data. For this purpose, we used tandem mass tag (TMT)-labeled samples from total cell lysates and secreted proteins from three different colorectal cancer cell lines. Following automated glycopeptide assignment (Byonic) and evaluation of the presence and relative intensity of oxonium ions, we observed that, in particular, the ratio of the ions at *m*/*z* 144.066 and 138.055, respectively, could be used to discriminate between *O*-GlcNAcylated and *O*-GalNAcylated peptides, with concomitant relative quantification between the different cell lines. Among the *O*-GalNAcylated proteins, we also observed anterior gradient protein 2 (AGR2), a protein which glycosylation site and status was hitherto not well documented. Using a combination of multiple fragmentation methods, we then not only assigned the site of modification, but also showed different glycosylation between intracellular (ER-resident) and secreted AGR2. Overall, our study shows the potential of broad application of the use of the relative intensities of oxonium ions for the confident assignment of glycopeptides, even in complex proteomics datasets.

## 1. Introduction

Nowadays, large-scale mass spectrometry (MS)-based proteomics is widely used in biomedical research, not only to investigate overall changes in protein abundance, for example by isotopic labeling approaches (e.g., SILAC, dimethyl labeling, and tandem mass tagging (TMT), but also to analyze post-translational modifications [1]. In order to reduce the complexity of the sample, enrichment of modified peptides/proteins is often applied prior to the downstream mass spectrometry analysis [2]. Moreover, unique characteristics of MS/MS fragmentation patterns, such as the presence of diagnostic ions, have also been exploited to aid in the analysis and confident identification of peptides in general, and post-translationally modified peptides in particular [3,4].

Also for the analysis of changes in protein glycosylation, MS analyses are now the gold standard. Glycomics approaches provide valuable information about the glycosylation status, either on an individual or defined group of proteins, or on full cellular scale. Alternatively, MS/MS analysis of intact glycopeptides can aid the identification of glycan structures in a protein specific manner, but multiple fragmentation techniques are usually required to determine site-specific (changes in) protein glycosylation. On the one hand, MS/MS spectra of glycopeptides containing a *N*-acetylhexosamine (HexNAc), as generally found in both *N*- and *O*-glycans, can be easily recognized by the presence of the corresponding oxonium ions in the low mass region of the spectra. On the other hand, their full characterization is far from trivial and several challenges for the assignment of glycopeptide MS/MS spectra are, amongst others, the dominance of glycosidic bond cleavages over peptide bond cleavages with most commonly applied fragmentation techniques, the complexity of potential glycan structures that can be present, long search times, especially for *O*-glycopeptides, and discrimination between isobaric structures. Hence, in the past decade there have been a lot of efforts to develop novel glycopeptide data acquisition methods [5,6,7] and suitable search algorithms [8,9,10]. Moreover, to simplify the glycan structures, glycoengineering of cellular systems [11], and glycosidases [12], have been used.

Notwithstanding, the discrimination between relatively simple glycopeptides, such as between *O*-GlcNAcylated peptides and *O*-GalNAcylated (Tn antigen containing peptides) is not straightforward, although tailored affinity purification steps can be helpful [13,14]. The discrimination between these two types of glycopeptides is, however, pivotal, given their very different biological roles [15,16]. Interestingly, it has previously been demonstrated that the relative intensity of different HexNAc oxonium ions can be used to discriminate between *O*-GlcNacylated peptides and Tn-glycopeptides [17]. This was initially investigated by testing fragmentation patterns of a set of synthetic glycopeptides and enriched sialylated peptides from urine and CSF [17]. Later, several other studies have also customized this idea in their glycoproteomics data analysis workflows [18,19,20]. However, the validity and applicability of such approaches could become stronger when it can also be used with large-scale proteomics datasets without a glycosylation focused approach, such as glycopeptide enrichment, during sample preparation, but this has hitherto not been demonstrated.

The aim of the current study was to investigate whether information on the presence and relative abundance of HexNAc oxonium ions can be used to scrutinize complex quantitative proteomics datasets (TMT-labeled peptides from three different cell lines) for the presence, and relative abundance, of specific glycopeptides. We show that especially for peptides containing an *O*-linked HexNAc, differentiation between an *O*-GlcNAc and *O*-GalNAc can be performed based on the relative intensity of just two oxonium ions. Following this approach, we demonstrate site specific, mucin-type, *O*-glycosylation of anterior gradient protein-2 (AGR2) and show that following secretion, AGR2 *O*-glycosylation is extended with more complex structures.

## 2. Results and Discussion

### 2.1. Differentiation of Glycopeptides Based on the Relative Abundance of Oxonium Ions

**Figure 1:**
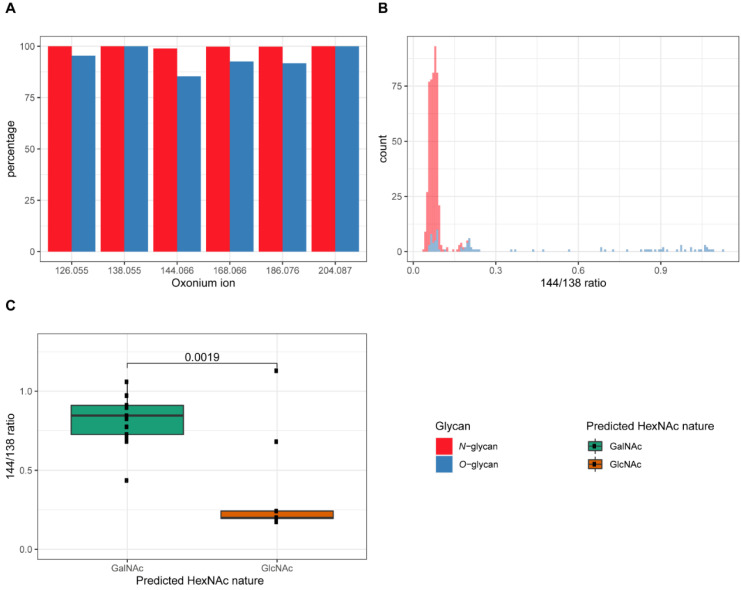
Discrimination of different glycopeptide populations. (**A**) Percentage (*y*-axis) of assigned *N*- (red) and *O*-glycopeptides (blue) spectra (with Byonic score above 250) containing the oxonium ions (*x*-axis). (**B**) Distribution of intensity ratio for oxonium ions at *m*/*z* 144.066 and 138.055 (=144/138) for *N*- and *O*-glycopeptides. The ratio was calculated when intensities from both oxonium ions were registered. (**C**) The box plot depicts the distribution of 144/138 ratios from unique *O*-glycopeptides based on their predicted single HexNAc nature (GalNAc versus GlcNAc). The HexNAc nature was predicted depending on the presence (for GalNAc, green) or absence (for GlcNAc, orange) of a transmembrane domain and/or a signal peptide. The prediction tools used were TMHMM Server v. 2.0, http://www.cbs.dtu.dk/services/TMHMM/ (accessed on 28 April 2021) and SignalP-5.0 Server, http://www.cbs.dtu.dk/services/SignalP/ (accessed on 28 April 2021), respectively. Data were statistically analyzed with Wilcoxon rank sum-test and *p* value is shown.

To speed up the database searches for glycopeptides, we have recently developed a tool that extracts MS/MS spectra based on the presence of the HexNAc oxonium ion at *m*/*z* 204.087 in the MS/MS data [21]. The specificity of this method is primarily determined by the *m*/*z* tolerance that is allowed during the selection of the spectra. With modern type, high-resolution, instruments the latter can be routinely set at 0.001 *m*/*z* units (i.e., 5 ppm). However, in addition to the above-mentioned ion, other related oxonium ions are generally present in glycopeptide spectra as well [7]. Using also the information from these ions could potentially make the selection more specific, which would be especially beneficial for complex datasets where no prior enrichment for glycopeptides was used. To test this, we examined LC-MS/MS data from TMT-labeled tryptic peptides from three different CRC cell lines (9-plex TMT, three cell lines in triplicate) acquired on an Orbitrap Exploris, prompted by the notion that the simultaneous quantification of the glycopeptide would be an additional asset. Moreover, we have an interest in these cell lines because we recently used them as a model system to study differential binding of a human lectin [22,23]. From the data, we selected MS/MS spectra with the fragment ion at *m*/*z* 204.087 and identified *N*- and *O*-glycopeptides using Byonic, accepting glycopeptide matches with a score above 250. Indeed, the other HexNAc oxonium ions in the low mass region were observed in almost all MS/MS spectra from these glycopeptides (Figure 1A), although it appeared that some *O*-glycopeptide MS/MS spectra lacked several oxonium ions.

To investigate whether approaches to use the relative abundance of oxonium ions to discriminate different populations of glycopeptides [17,18,19,20] can also be applied to our dataset, we analyzed the filtered data for the ratios of several of the oxonium ions and observed that especially the ratio of the oxonium ions at *m*/*z* 144.066 and 138.055, respectively, showed potential to discriminate different sets of glycopeptides (Figure 1B).

**Figure 2:**
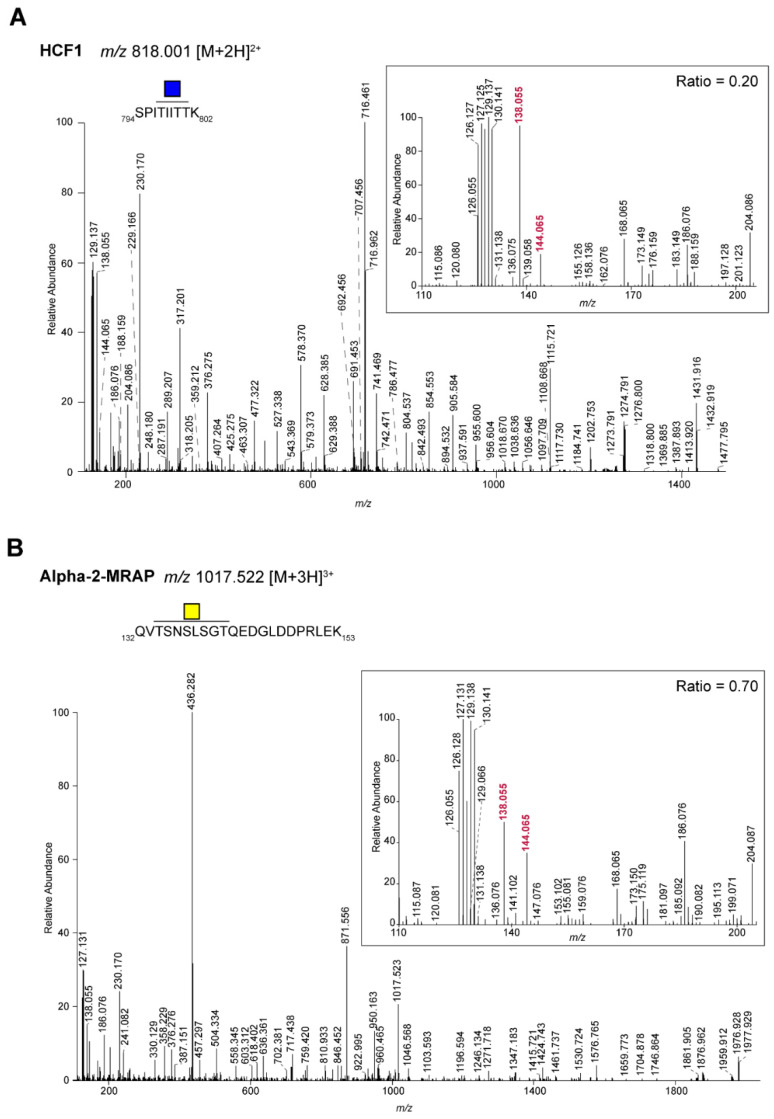
MS/MS spectra of TMT-labeled tryptic *O*-glycopeptides from CRC cell lines. HCD MS/MS spectra of an *O*-GlcNacylated (**A**), Host cell factor 1 (HCF1)) and *O*-GalNacylated (**B**), alpha-2-macroglobulin receptor-associated protein (Alpha-2-MRAP)) TMT-labeled tryptic peptide. Blue square: GlcNAc; yellow square: GalNAc. The inserts show the TMT-reporter (9-plex), oxonium ions and ratio of *m*/*z* 144.066/138.055. The oxonium ions at *m*/*z* 138.055 and 144.066 used for the ratio are shown in red. Samples were labeled as follows: HT29: 126, 127N,127C; HCT116: 128N, 128 C, 129N; LS174T: 129C, 130N, 130C. Of note, *m*/*z* 126.055 represents the oxonium ion [HexNAc-C_2_H_6_O_3_]^+^.

**Figure 3:**
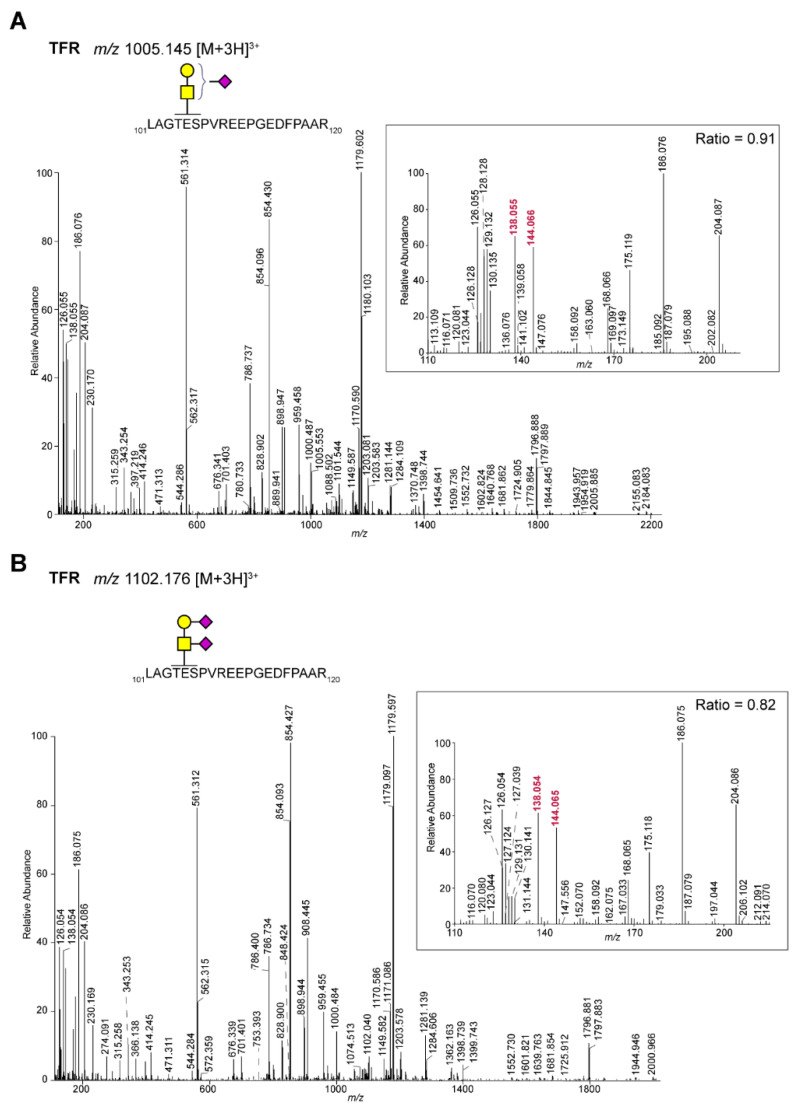
MS/MS spectra of a TMT-labeled tryptic peptide from human transferrin receptor (TFR) carrying a mono and di-sialylated T-antigen. TMT-labeled tryptic peptides form CRC cell lines were analyzed by LC-MS/MS using higher-energy collisional dissociation. The tryptic peptide LAGTESPVREEPGEDFPAAR from TFR was observed with a mono- (**A**) and di-sialylated (**B**) T antigen, respectively. In both spectra a high intensity of the oxonium ion at *m*/*z* 144.066 versus 138.055 was observed. The inserts show the TMT reporter (9-plex), oxonium ions and ratio of *m*/*z* 144.066/138.055. Samples were labeled as follows: HT29: 126, 127N, 127C; HCT116: 128N, 128 C, 129N; LS174T: 129C, 130N, 130C. Of note, *m*/*z* 126.055 represents the oxonium ion [HexNAc-C_2_H_6_O_3_]^+^. Yellow square: GalNAc; yellow circle: galactose; purple diamond: *N*-acetylneuraminic acid. The oxonium ions at *m*/*z* 138.055 and 144.066 used for the ratio are shown in red.

For the spectra from the identified *N*-glycopeptides (Figure 1B, Table S1), the ion at *m*/*z* 144.066 was always much lower than the one at *m*/*z* 138.055. On the other hand, a population of MS/MS spectra from *O*-glycopeptides (Figure 1B, Table S1) was found where the intensity of the ion at *m*/*z* 144.066 was higher than that at *m*/*z* 138.055. Within the set of identified *O*-glycopeptides, we thought to focus on peptides with a single HexNAc because they potentially show the cleanest GalNAc vs. GlcNAc ratio of the oxonium ions (Table 1). For the well-known *O*-GlcNAcylated protein host cell factor 1 (HCF 1), the *O*-glycopeptides show a very low ratio (<0.3), while for clear membrane proteins, e.g., transferrin receptor protein 1, the ratio was much higher. Since we do not have independent information from literature or other sources about the nature of the HexNAc for all the proteins in Table 1, we bioinformatically assigned this as either a GalNAc (signal peptide and/or transmembrane domain present) or GlcNAc (signal peptide and/or transmembrane domain absent). Based on this information, we plotted the 144.066/138.055 ratio (Figure 1C) for the two populations, which clearly showed the difference in the ratios. However, for O-GlcNAcase, which was predicted to contain a GlcNAc, clearly showed a ratio resembling a GalNAc. Hence, evidence from independent analyses is needed to determine the nature on the HexNAc on this protein. As examples, Figure 2A,B show the spectra of a peptide with an *O*-GlcNAc (host cell factor 1 tryptic peptide) and *O*-GalNAc (alpha-2-macroglobulin receptor-associated protein), respectively. The high ratio of the 144.066/138.055 ions in peptides with a single *O*-GalNAc was also found in peptides with sialylated or di-sialylated T-structures. For example, in Figure 3, the mono and di-sialylated T peptides LAGTESPVREEPGEDFPAAR (potential glycosylation sites indicated in red) from the human transferrin receptor are shown (Uniprot ID: P02786). Because we used TMT-labeled peptides, we could in parallel see that the di-sialylated peptide was more abundant in the HT29 cells, while the mono-sialylated was higher in the LS174T and HCT116 cells.

Overall, the above data show that even from complex datasets, specific *O*-glycopeptides with *O*-linked GlcNAc versus GalNAc can be discriminated by the presence and relative abundance of several oxonium ions.

### 2.2. Site-Specific Identification of the O-glycosylation of Anterior Gradient Protein 2 (AGR2)

**Figure 4:**
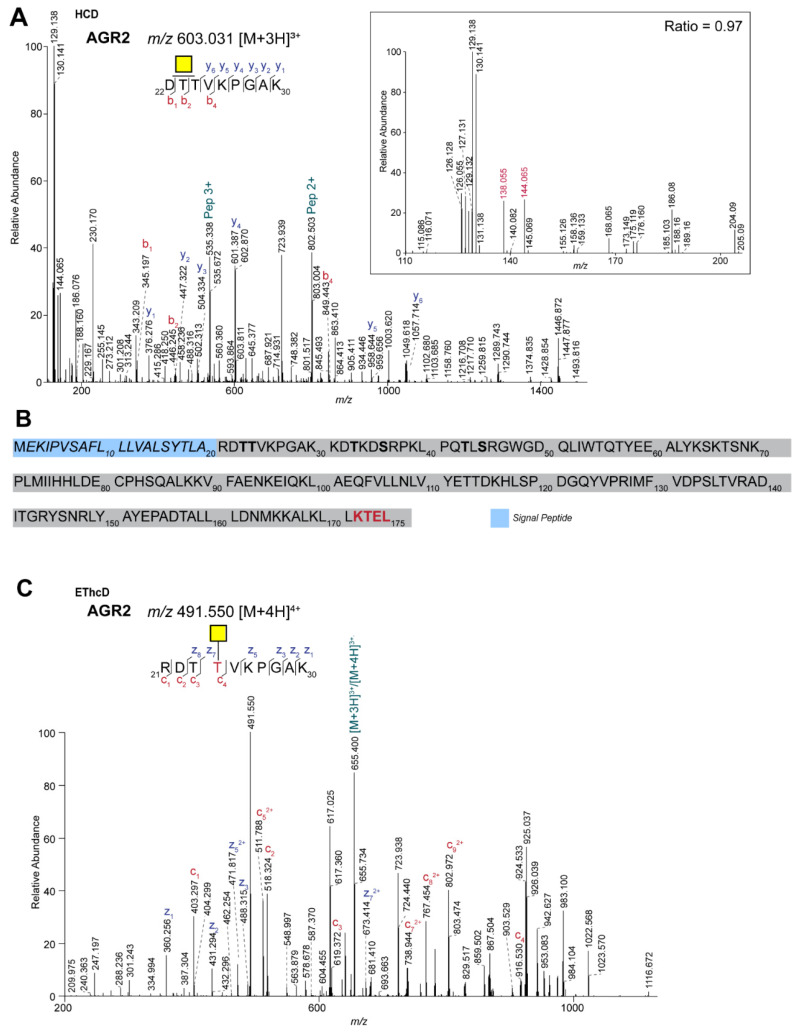
Site-specific identification of *O*-GalNAcylation of anterior gradient protein 2 (AGR2) in CRC cell lines. (**A**) HCD spectrum of the AGR2 TMT-labeled tryptic peptide DTTVKPGAK carrying a Tn antigen. The insert shows the TMT-reporter (9-plex), oxonium ions and ratio of *m*/*z* 144.066/138.055. The oxonium ions at *m*/*z* 138.055 and 144.066 used for the ratio are shown in red. Samples were labeled as follows: HT29: 126, 127N,127C; HCT116: 128N, 128 C, 129N; LS174T: 129C, 130N, 130C. Of note, *m*/*z* 126.055 represents the oxonium ion [HexNAc-C_2_H_6_O_3_]^+^.Yellow square: GalNAc; Pep: peptide. (**B**) AGR2 protein sequence. The signal peptide is highlighted in blue and italic. In bold, predicted glycosylation sites are highlighted [25]. In red, the ER retention signal KTEL is indicated. (**C**) EThCD spectrum of the *O*-GalNAcylated TMT-labeled tryptic peptide RDTTVKPGAK from AGR2.

One of the identified peptides with a single HexNAc (Supplemental Table S1) corresponds to the tryptic peptide DTTVKPGAK from anterior gradient protein 2 (AGR2, Uniprot ID O95994). Although it has been demonstrated that this protein can be *O*-glycosylated [24], the identity of the glycan remained elusive. Moreover, since the initial discovery of AGR2 *O*-glycosylation, there has been confusion about the nature of the HexNAc residue and studies of its *O*-GlcNAcylation have also been performed [25]. Based on the ratio of the ions at *m*/*z* 144.066 and 138.055 as observed in the HCD MS/MS spectrum of the AGR2 tryptic peptide (Figure 4A), we provide strong evidence that, at least in the CRC cells, AGR2 is modified with a single *O*-GalNAc. Of note, the AGR2 *O*-glycopeptide was found at higher levels in one of the three cell lines (LS174T) compared to the others (HT29 and LS174T). This corresponds to the overall higher level of AGR2 that was found in this cell line [23], indicating that the AGR2 *O*-glycosylation per se is not different between the CRC cell lines.

The peptide on which the *O*-GalNAc was observed is located close to the endogenous N-terminus of the protein, after removal of the signal peptide (Figure 4B). This mature form of AGR2 starts with an N-terminal arginine. Interestingly, within our data, we also observed the missed cleaved peptide RDTTVKPGAK modified with a single HexNAc showing the specific ratio of the ions at *m*/*z* 144.066 and 138.055 in the fragmentation spectrum (data not shown). In fact, the intensity of this peptide within our data was even higher than the fully tryptic peptide described above, probably reflecting poor exoproteolytic activity for trypsin.

Previously, a bioinformatic approach was used to predict the site of AGR2 *O*-glycosylation [24] and the peptide that we identified covered two threonines of the six predicted sites (4 Thr, 2 Ser, Figure 4B). In order to unambiguously demonstrate the *O*-glycosylation site on AGR2, we performed EThcD experiments. Because higher charged precursor ions are preferred for this type of analyses, and the fact that the ion was found at higher intensities, we decided to focus on the quadruply charged ion at *m*/*z* 491.550 (Figure 4C, RDTTVKPGAK+1 GalNAc, [M+4H]^4+^). Based on the presence of the c_3_ and z_7_^2+^ ions at *m*/*z* 619.372 and 673.414, respectively, we concluded that the GalNAc is present on Thr-4 of this peptide. This was supported by the absence of the ions at *m*/*z* 822.452 and 571.875, which would correspond to these ions under the scenario that Thr-3 was *O*-glycosylated.

In cells, AGR2 is primarily found in the ER, due to the presence of a KDEL-like ER retention signal (KTEL, Figure 4B) at the C-terminus. However, several studies have shown that AGR2 can also be secreted [24,25], and it was shown by western blot analysis that secreted AGR2 had an apparent molecular weight of approximately one kilodalton higher than intracellular AGR2. Our current data provide evidence for *O*-glycosylation of intracellular AGR2, which is in line with a recent study that demonstrated similar *O*-glycosylation of calnexin, another member of ER-resident oxidoreductases [26]. However, it leaves open the possibility of differential glycosylation of secreted versus ER-resident AGR2. Hence, to get further insight into secreted AGR2, and its *O*-glycosylation, we analyzed the secreted proteins from the same three cell lines (LS174T, HT29, and HCT116) in a TMT labeling experiment. Overall, we identified more than 1500 proteins (with at least three unique peptides) and interestingly, although based on a single biological replicate, AGR2 had a higher abundance in LS174T than in the other two cell lines. This is in line with AGR2 abundance in the total cell lysate as described above, although the secreted AGR2 ratio was much higher (LS174T/HCT116 = 17, LS174T/HT29 = 10). Among other proteins that were found at higher levels in the secretome of the LS174T cells was AGR3, but also several mucins. The latter is consistent with the higher mucus production that had previously been demonstrated for this cell line [27]. Of note, AGR2 itself plays an essential role in mucus production [24,28].

### 2.3. Differential Glycosylation of Secreted Versus Intracellular AGR2

**Figure 5:**
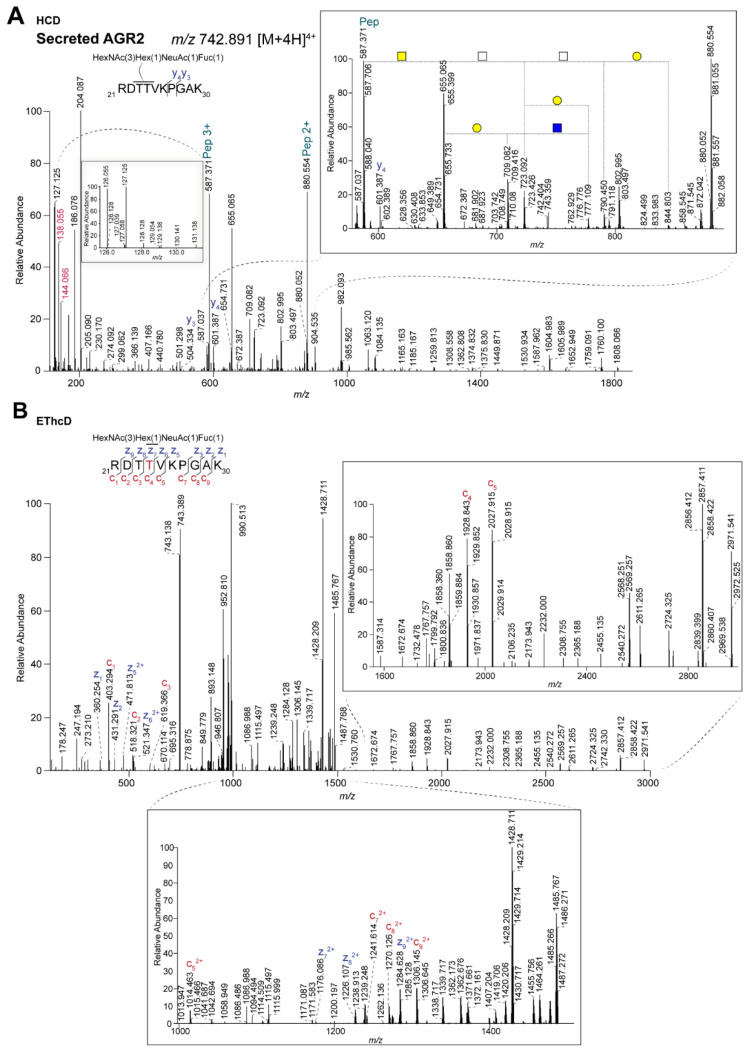
Identification of larger *O*-glycan structures on secreted AGR2. (**A**) HCD and (**B**) EThcD spectra of the TMT-labeled tryptic peptide RDTTVKPGAK from secreted AGR2, observed at *m*/*z* 742.891 [M+4H]^4+^, carrying a glycan composed of HexNAc(1)Hex(3)NeuAc(1)Fuc(1). Pep: peptide; Blue square: GlcNAc; yellow square: GalNAc; yellow circle: galactose. The inserts represent a zoom in of the spectra from *m*/*z* 580–890 (**A**), representing glycan fragmentation) and *m*/*z* 1000–1500 (**B**). c and z ions are represented in red and blue, respectively. Thr-4 (in red within the peptide sequence) indicates the glycosylation site.

To gain insight in the glycosylation of secreted AGR2, we searched the secretome data for AGR2 *O*-glycopeptides. First of all, the tryptic AGR2 peptide RDTTVKPGAK with the single GalNAc, as described above was again found. In addition, we also observed this same peptide with a more complex glycan structure (Figure 5A, H1N3F1S1) at *m*/*z* 742.891 ([M+4H]^4+^. Although different cells may have different glycosylation, the size of this glycan nicely corresponds to the difference in apparent molecular weight of intracellular and secreted AGR2, as mentioned above. Based on *O*-glycan structures that have been described in these cell lines, [29], especially LS174T where the highest signal for this peptide was observed, we initially thought the composition most probably corresponds to a core 4 structure. The combination of both GalNAc and GlcNAc residues in such a structure could explain the intermediate *m*/*z* 144.066/138.055 ion ratio of 0.5 that was observed in the corresponding MS/MS spectrum, highlighting the difficulty for using the oxonium ion ratios for more complex glycans. However, some features of the MS/MS spectrum are not compatible with a core 4 structure (GlcNAcβ1-3(GlcNAcβ1-6)GalNAc-Ser/Thr). For example, it contains ions corresponding to the peptide+HexNAc+Hex (*m*/*z* 709.082, 3+) and peptide+3HexNAc (*m*/*z* 790.450, 3+), respectively.

**Figure 6:**
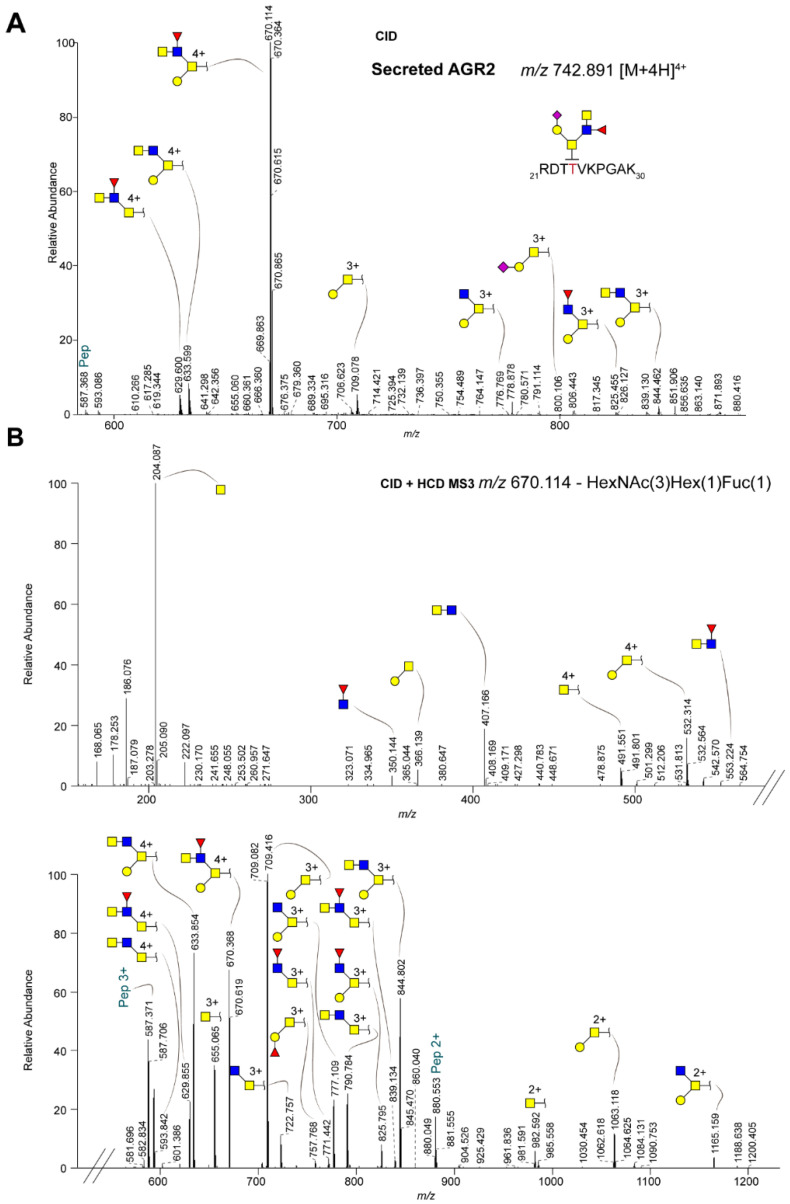
In-depth MS characterization of the glycan structure on secreted AGR2. (**A**) The TMT-labeled AGR2 tryptic *O*-glycopeptide from secreted AGR2 (see Figure 5B), was analyzed after CID fragmentation. The proposed glycan structure on secreted AGR2 is shown on the peptide sequence. (**B**) The ion representing the loss of the terminal sialic acid (*m*/*z* 670.114) was subsequently selected for HCD fragmentation (upper and lower panel, representing different regions of the resulting MS/MS spectrum). The terminal end of the glycan structures indicates the TMT-labeled tryptic peptide RDTTVKPGAK from secreted AGR2. Blue square: GlcNAc; yellow square: GalNAc; yellow circle: galactose; red triangle: fucose; purple diamond: *N*-acetylneuraminic; Pep: peptide.

To explain these results, we hypothesized that, in contrast to intracellular AGR2, secreted AGR2 could be glycosylated on both threonines within the RDTTVKPGAK peptide, each with a different small *O*-glycan structure. To test this hypothesis, we used EThcD fragmentation, which showed the full glycan structure only on Thr-4 in the RDTTVKPGAK peptide, thereby excluding the two-site occupancy option (Figure 5B). Second, one may speculate about possible glycan rearrangements during high energy HCD fragmentation that was used to generate good peptide fragmentation and high TMT reporter ion intensities or that secreted AGR2 is modified with different isobaric structures which co-elute under our experimental conditions. To get more insight about the glycan structure on secreted AGR2, we performed additional mass spectrometry experiments (Figure 6). Following CID fragmentation, the precursor primarily lost the terminal sialic acid, resulting in a major ion at *m*/*z* 670.114 ([M+4H]^4+^). When this ion was subsequently selected for MS^3^ using HCD fragmentation with a normalized collision energy of 30%, additional losses of the monosaccharides were observed (Figure 6B). Based on these spectra, especially the 4+ charged ions showing the loss of either the fucose (*m*/*z* 633.599) or hexose (*m*/*z* 629.600), the most likely glycan structure is a core 2 with a fucosylated LacdiNAc (LDN-F). This is supported by several oxonium ions that were detected in the lower mass region of these spectra, e.g., at *m*/*z* 350.144, 366.139, 407.166, and 553.224. Of note, although based on the data we cannot rule out the possibility that the GalNAc in the LDN structure is fucosylated, we deem this option highly unlikely since—to our knowledge—there is no human glycosyltransferase that could catalyze such a reaction. We also observed an ion at *m*/*z* 757.788 ([M+3H]^3+^), representing a fragment with a fucosylated T antigen. We think fucose transfer during fragmentation is the most plausible explanation for this. Next, we screened our data for the presence of shorter glycan structures that may further substantiate our assignment, but apart from the peptide with the single GalNAc, these were not observed. Importantly though, the peptides with either the single GalNAc or the H1N3F1S1 glycan were well-separated (Supplemental Figure S1), showing that the former was not due to in source fragmentation. Notwithstanding, we consider it likely that the peptide with the single GalNAc that we observed in the secretome data is due to contamination of intracellular material, as is commonly observed in secretome analyses, and was also apparent from the overall list of identified proteins that we identified in the secretome analysis.

Overall, following the initial discovery of AGR2 glycosylation based on our analysis of oxonium ions in spectra from quantitative proteomics experiment, the above data show that intracellular AGR2 is *O*-glycosylated with a single GalNAc at Thr-4 of the mature protein, but upon secretion also larger *O*-glycan structures are found. In conclusion, our study demonstrates how signatures of glycopeptide fragmentation spectra can reveal new insights in protein specific glycosylation, even in complex quantitative mass spectrometry datasets.

## 3. Materials and Methods

### 3.1. Cell Lines Culture

HCT116 and HT29 cell lines were obtained from the Department of Surgery of the Leiden University Medical Center (Leiden, The Netherlands), whereas LS174T were provided by the Amsterdam UMC (Amsterdam, the Netherlands). Short tandem repeat (STR) profiling was used for cell line authentication at the forensic laboratory for DNA research (ISO 17025), all showing 100% match with the known profile [30]. All cell lines were maintained at 5% CO2 and 37 °C in RPMI-1640 medium supplemented with L-glutamine, 10% fetal bovine serum (FBS) (Invitrogen, Carlsbad, CA, USA), and streptomycin/penicillin (Sigma-Aldrich, St. Louis, MO, USA). For harvesting, cells were washed twice with 1× PBS before incubation for approximately 5 min in 1× trypsin/EDTA solution in 1× PBS. Then, trypsin’s activity was inhibited by the addition of serum containing medium. Cells were subsequently collected and counted using the CountessTM Automated Cell Counter (Invitrogen, Paisley, UK). Aliquots of 4 × 10^6^ cells were washed with 1× PBS, centrifuged at 1500 rpm and obtained cell pellets were stored at −20 °C until use for TMT labeling.

### 3.2. Secretome Collection

Cells were grown until 70% of confluence in a T175 cm^2^ flask in medium containing 10% FBS as described above. Then, cells were washed three times with 1x PBS solution and once with medium without FBS supplementation, followed by 1 h pre-incubation in serum-free media. Subsequently, the media was discarded and cells were incubated in serum-free media for 24 h. Conditioned media (CM) were centrifuged at 300g for 10 min and subsequently passed through a 0.45 µm filter (Millex-HV, Millipore, Amsterdam, The Netherlands) to remove cell debris. Finally, the CM was enriched in protein content using 10 K MWCO centrifugal concentrator (Amicon ultra- 4 °C, Millipore, Amsterdam, The Netherlands), according to product information. Bradford assay (Thermo Fisher Scientific, Waltham, MA, USA) was used for protein quantification and samples were frozen at −80 °C until use.

### 3.3. TMT Labeling and LC-MS/MS Analyses

Cell lysis, digestion and TMT labeling was performed as described before [22]. Briefly, cellular pellets from HCT116, HT29, and LS174T cells were used for protein extracts, in triplicate and 100 µg of protein was used for subsequent reduction with TCEP (5 mM), alkylation with iodoacetamide (15 mM) and quenching with 10 mM DTT. Methanol-chloroform precipitation was used to obtain cleaned protein pellets, then resuspended in 40 mM HEPES (pH 8.4) to perform trypsin (10 µg) digestion O/N at 37 °C. Ten µg of each of the samples was labelled with one of the nine amino reactive TMT10plex Label Reagents (HT29: 126, 127N,127C, HCT116: 128N, 128 C, 129N, LS174T: 129C, 130N, 130C), Thermo Scientific, lot no. UG282327) for 1 h at RT and excess TMT label was quenched by 5% hydroxylamine for 15 min at RT. The labeled peptide samples were then mixed, fractionated by high-pH SPE in five fractions, freeze-dried and stored at −20 °C prior to LC-MS/MS analysis. For the secretome samples, 10 µg of secreted proteins from the three different cell lines was processed as described above in a triplex experiment (LS174T: 127N, HCT116: 129C, HT29: 131).

TMT-labeled peptides were analyzed by online C18 nano-HPLC MS/MS coupling an Ultimate 3000 HPLC system (Thermo, Bremen, Germany), and an Orbitrap Exploris™ 480 mass spectrometer (Thermo). Fractions were injected onto a homemade precolumn (100 µm × 15 mm; Reprosil-Pur C18-AQ 3 µm, Dr Maisch, Ammerbuch, Germany), equilibrated with solvent A (100/0.1 water/formic acid *v*/*v*) and eluted via a homemade analytical nano-HPLC column (50 cm × 75 µm; Reprosil-Pur C18-AQ 1.9 µm) using solvent B (80/20/0.1 acetonitrile/water/formic acid *v*/*v*/*v*). For the cellular proteins, the gradient was from 5–30% B in 240 min and samples were analysed in duplicate, for the secreted proteins samples were analyzed once using a gradient from 2–36% B in 120 min. The MS spectra were recorded at *m*/*z* range 350–1200 in the Orbitrap at a resolution of 120,000. Dynamic exclusion was after n = 1 with an exclusion duration of 45 s and a mass tolerance of 10 ppm. Precursors were fragmented by high energy collision-induced dissociation (HCD, normalized collision energy of 36%) using the Orbitrap.

For EThcD (ETD 29.06 and HCD 15.00) and MS3 analysis (combining CID and HCD) samples were analyzed on an Orbitrap Fusion Lumos mass spectrometer coupled to an Easy nLC 1000 gradient HPLC system (Thermo) using targeted analysis of selected precursors. The same columns and solvents as mentioned above were used and the gradient was from 10–40% B in 60 min.

### 3.4. Bioinformatic Workflow for Glycopeptides Identification

Fragmentation spectra containing the HexNAc oxonium ion at *m*/*z* 204.087 were searched for glycopeptides against the human database (20596 entries, Feb 2019) using Byonic version 2.13.2 (proteinmetrics.com). Two glycan databases consisting of 309 common mammalian *N*-glycans (for *N*-glycopeptide searches) and 70 human *O*-glycans (for *O*-glycopeptide searches) were used, respectively. Trypsin was selected as enzyme and two missed cleavages were allowed. A precursor and fragment tolerance of 10 and 20 ppm, respectively, was allowed. For both *N*- and *O*-glycopeptide searches, glycosylation was set as rare modification, while oxidation of Met was set as a common, dynamic modification. The maximum number of common and rare modifications were set at 2 and 1, respectively. Carbamidomethyl (Cys) and TMT-10 plex (+229.162932, on Lys and peptide N-terminus) were set as static modifications. A Byonic score higher than 250 was considered stringent for further glycopeptides selection. Parallel to the search, the presence and intensity of oxonium ions at *m*/*z* 126.055 [HexNAc-C_2_H_6_O_3_]^+^, 138.055 [HexNAc-CH_6_O_3_]^+^, 144.066 [HexNAc-C_2_H_4_O_2_]^+^, 168.066 [HexNAc-2H_2_O]^+^, 186.076 [HexNAc-H_2_O]^+^, 204.087 [HexNAc]^+^, were registered with a tolerance of *m*/*z* 0.001, minimum intensity of 1000 count, and a peak rank in the top 100 peaks (in house software, available at http://cpm.lumc.nl/yassene/oxonium-ions-filter/, accessed on 28 April 2021). Finally, the peptide identifications with scores 250 and higher (Byonic search) and the oxonium ions intensities were linked according to the spectrum ID and merged into one dataset for further processing. For the prediction of transmembrane helices in proteins, TMHMM Server v. 2.0, http://www.cbs.dtu.dk/services/TMHMM/ (accessed on 28 April 2021) was used. SignalP-5.0 Server, http://www.cbs.dtu.dk/services/SignalP/ (accessed on 28 April 2021) was used for the prediction of signal peptides.

### 3.5. Quantitative Proteomic Data Analysis of Secreted Proteins

For peptide identification, MS/MS spectra were searched against the human database (20596 entries, Feb 2019) using Mascot Version 2.2.07 (Matrix Science) with the following settings: 10 ppm and 20 milli mass units deviation for precursor and fragment masses, respectively. Trypsin was set as enzyme and two missed cleavages were allowed. Carbamidomethyl on cysteines and TMT6plex on Lys and N-term were set as fixed modifications. Variable modifications were oxidation (on Met) and acetylation on the protein N-terminus. All searches and subsequent data analysis, including Percolator [31] and relative quantification, were performed using Proteome Discoverer 2.4 (Thermo Scientific). Peptide-spectrum matches were adjusted to a 1% FDR.

## Figures and Tables

**Table 1:** Discrimination of *O*-glycopeptides with a single HexNAc based on the intensities of the oxonium ions at *m*/*z* 144.066 and 138.055 (144/138 ratio).

Uniprot ID	Protein Name	Peptide Sequence	Glycan	Byonic Score	144/138 Ratio	Predicted * HexNAc Nature
Q12830	Nucleosome-remodeling factor subunit BPTF	TVITEVTTMTSTVATESK	N_1_	457	0.17	GlcNAc
Q96HC4	PDZ and LIM domain protein 5	EVVKPVPITSPAVSK	N_1_	508	0.18	GlcNAc
P51610	Host cell factor 1	TIPMSAIITQAGATGVTSSPGIK	N_1_	1001	0.19	GlcNAc
P51610	Host cell factor 1	SPITIITTK	N_1_	664	0.20	GlcNAc
Q6UN15	Pre-mRNA 3’-end-processing factor FIP1	ETALPSTK	N_1_	362	0.20	GlcNAc
P02545	Prelamin-A/C	ASASGSGAQVGGPISSGSSASSVTVTR	N_1_	842	0.20	GlcNAc
P27816	Microtubule-associated protein 4	ASPSKPASAPASR	N_1_	309	0.24	GlcNAc
Q8NBJ4	Golgi membrane protein 1	LQAAGLPHTEVPQGK	H_1_N_1_S_2_	619	0.44	GalNAc
Q92753	Nuclear receptor ROR-beta	QQQSGEAEALAR	H_1_N_1_S_2_	273	0.68	GlcNAc
P14314	Glucosidase 2 subunit beta	SEALPTDLPAPSAPDLTEPK	H_1_N_1_S_2_	775	0.68	GalNAc
P30533	Alpha-2-macroglobulin receptor-associated protein	QVTSNSLSGTQEDGLDDPRLEK	N_1_	575	0.70	GalNAc
Q8IYS2	Uncharacterized protein KIAA2013	VAALQTVGPTAGPAPK	H_1_N_1_S_2_	695	0.73	GalNAc
Q8NBS9	Thioredoxin domain-containing protein 5	TETGATETVTPSEAPVLAAEPEADK	N_1_	609	0.77	GalNAc
P02786	Transferrin receptor protein 1	LAGTESPVREEPGEDFPAAR	H_1_N_1_S_2_	364	0.82	GalNAc
P02786	Transferrin receptor protein 1	LAGTESPVREEPGEDFPAAR	H_1_N_1_F_1_	406	0.85	GalNAc
Q10469	Alpha-1,6-mannosyl-glycoprotein 2-beta-N-acetylglucosaminyltransferase	GGDHPSVAVGIR	H_1_N_1_S_2_	456	0.90	GalNAc
P02786	Transferrin receptor protein 1	LAGTESPVREEPGEDFPAAR	H_1_N_1_S_1_	596	0.91	GalNAc
P26572	Alpha-1,3-mannosyl-glycoprotein 2-beta-N-acetylglucosaminyltransferase	GRVPTAAPPAQPR	H_1_N_1_S_2_	260	0.91	GalNAc
Q92520	Protein FAM3C	STKPPR	H_1_N_1_S_2_	285	0.97	GalNAc
O95994	Anterior gradient protein 2 homolog	DTTVKPGAK	N_1_	376	0.97	GalNAc
P14314	Glucosidase 2 subunit beta	SEALPTDLPAPSAPDLTEPK	N_1_	674	1.06	GalNAc
O60502	Protein O-GlcNAcase	QVAHSGAK	N_1_	374	1.13	GlcNAc

The overall list of *O*-glycopeptides was filtered based on a Byonic score above 250 and HexNAc(1) (N_1_) containing glycans. The resulting proteins are shown, with Uniprot identification number (ID), protein name, peptide sequence, glycan composition (N=HexNAc, H=Hexose, S=Sialic acid, F=Fucose), Byonic score, ratio of 144.066/138.055 oxonium ions and predicted HexNAc nature. *: the HexNAc nature was predicted depending on the presence (for GalNAc) or absence (for GlcNAc) of a transmembrane domain and/or a signal peptide. The prediction tools used were TMHMM Server v. 2.0, http://www.cbs.dtu.dk/services/TMHMM/ (accessed on 28 April 2021) and SignalP-5.0 Server, http://www.cbs.dtu.dk/services/SignalP/ (accessed on 28 April 2021), respectively.

## Data Availability

The mass spectrometry proteomics data have been deposited to the ProteomeXchange Consortium via the PRIDE [32] partner repository with the dataset identifier PXD025805.

## Supplementary Materials

The following is available online at https://www.mdpi.com/article/10.3390/ijms22105369/s1.
