# Supplementary material for: Oxonium Ion Guided Analysis of Quantitative Proteomics Data Reveals Site-Specific O-Glycosylation of Anterior Gradient Protein 2 (AGR2)
Source: Int J Mol Sci. 2021 May 20;22(10):5369. doi: 10.3390/ijms22105369 (PMC8160981; doi:10.3390/ijms22105369)
Supplement: Supplementary file 1 [file ijms-22-05369-s001.zip › Figure SI.pdf]

Supplementary Materials

Oxonium Ion Guided Analysis of Quantitative Proteomics Data Reveals Site-Specific *O*-glycosylation of Anterior Gradient Protein 2 (AGR2)

Martina Pirro [1], Yassene Mohammed [1,2], Arnoud H. de Ru [1], George M. C. Janssen [1], Rayman T. N. Tjokrodirijo [1], Katarina Madunić [1], Manfred Wuhrer [1], Peter A. van Veelen [1] and Paul J. Hensbergen [1,*]

[1] Center for Proteomics and Metabolomics, Leiden University Medical Center, 2333 ZA, Leiden, The Netherlands; M.Pirro@lumc.nl (M.P.); Y.Mohammed@lumc.nl (Y.M.); A.H.de_Ru@lumc.nl (A.H.d.R.); G.M.C.Janssen@lumc.nl (G.M.C.J.); R.T.N.Tjokrodirijo@lumc.nl (R.T.N.T.); K.Madunic@lumc.nl (K.M.); M.Wuhrer@lumc.nl (M.W.); P.A.van_Veelen@lumc.nl (P.A.v.V.)

[2] Genome BC Proteomics Centre, University of Victoria, Victoria, BC V8Z 7X8, Canada

* Correspondence: P.J.Hensbergen@lumc.nl; Tel.: +31-71-5266394; Fax: +31-71-5266907

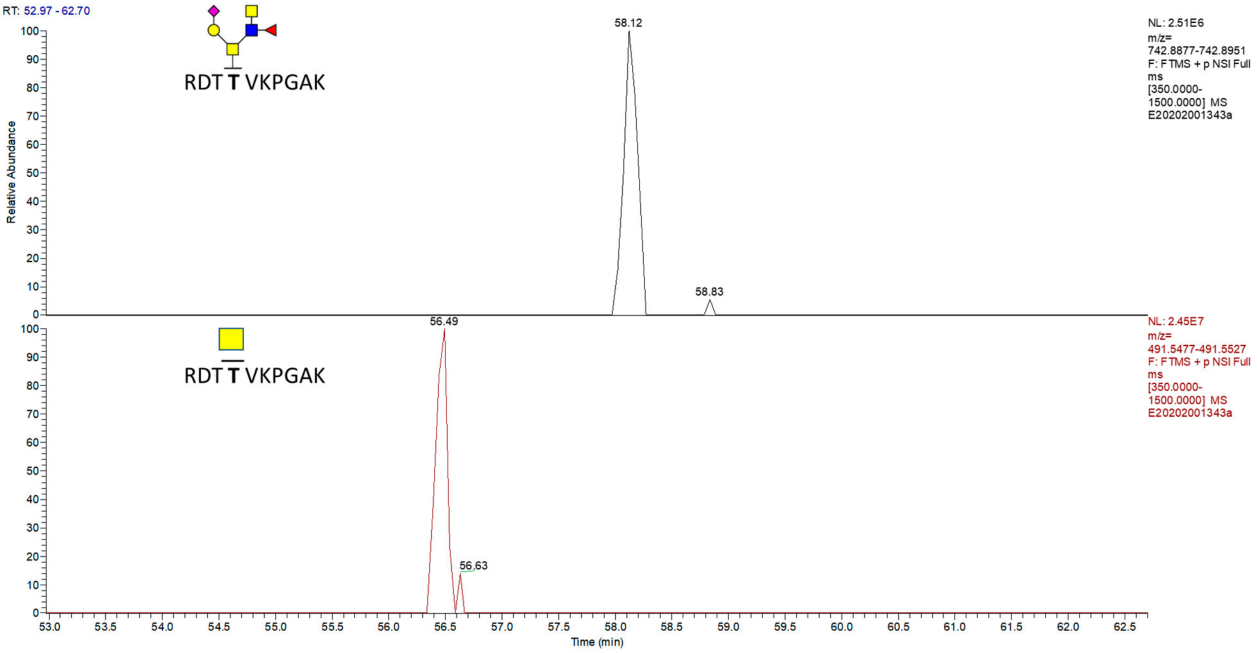

Figure S1. Separation of AGR2 glycopeptides. Chromatographic elution profile of the AGR2 tryptic peptide RDTTVKPGAK carrying the H1N3F1S1 glycan (upper panel, *m*/*z* 742.891, RT: 58.12) or the single GalNAc (lower panel, *m*/*z* 491.550, RT: 56.49). Blue square: GlcNAc; yellow square: GalNAc; yellow circle: galactose; red triangle: fucose; purple diamond: *N*-acetylneuraminic acid.
